# Effect of mild brain traumatic injury on intelligence and memory function in motorcycle riders 

**Published:** 2019-08

**Authors:** Elham Shafiei, Arash Nademi, Mahtab Bonyadi, Esmaeil Fakharian

**Affiliations:** ^*a*^Clinical Reasearch Development Unit, Mostafa KHomeini Hospital, Ilam University of Medical Science, Ilam, Iran.; ^*b*^Department of Statistics, Ilam Branch Islamic Azad University, Ilam, Iran.; ^*c*^Trauma Research Center, Kashan University of Medical Science, Kashan, Iran.

## Abstract

**Background:**

The most common causes of traumatic brain injury are vehicle crashes, including motorcycles, which lead to long-term disabilities. The purpose of this study was to investigate the effect of mild brain trauma on intelligence and memory function in motorcycle riders suffering from mild tumor injury.

**Methods:**

In this prospective cohort study, intelligence and memory functions of 87 motorcyclists suffering from mild traumatic brain injury were investigated at the beginning of the study (M=0, IQ=0) and six months after the trauma (M=6, IQ=6) using Wechsler intelligence and memory scale. Moreover, the obtained results were compared with those of 87 normal people. Data were analyzed using ANCOVA, the Chi-square and independent t-test.

**Results:**

The mean score of the Wechsler memory scale in motorcycle riders with mild traumatic brain injury were similar to those of normal subjects at the beginning of the study. However, this score was lower in traumatic brain injury patients six months after the trauma, compared to normal people. Moreover, the obtained results showed a significant difference between the intelligence function of traumatic brain injury patients and that of normal people only six months after the trauma (P≤0.05).

**Conclusions:**

According to the results, patients with mild traumatic memory and conceptualization are slightly weaker than normal patients. In addition, there was a significant difference between the patients and normal people regarding the intelligence function. Cognitive rehabilitation is one of the necessary psychological interventions for the treatment of patients with traumatic brain injuries.

**Keywords:**

Functional memory, Intelligence, Motorcycle, Traumatic brain injury

